# LAPTM4B in tumor immune evasion: lysosomal autophagy, metabolic adaptation, and therapeutic resistance

**DOI:** 10.3389/fimmu.2026.1946009

**Published:** 2026-09-30

**Authors:** Haokun Leng, Zetong Zheng, Ruizheng Li, Qi Yuan, Siheng Lv, Dong Yan, Lei Wu

**Affiliations:** 1 Beijing Luhe Hospital, Capital Medical University, Beijing, China; 2 School of Public Health, Capital Medical University, Beijing, China; 3 Cancer Center, Beijing Luhe Hospital, Capital Medical University, Beijing, China

**Keywords:** autophagy, immunometabolism, LAPTM4B, therapeutic resistance, tumor immune evasion, tumor microenvironment

## Abstract

**Background:**

Tumor immune evasion is shaped not only by canonical immune checkpoints but also by lysosomal stress adaptation, autophagy, metabolic remodeling, and therapy-induced survival programs within malignant cells. Lysosomal-associated protein transmembrane 4 beta (LAPTM4B), an oncogenic lysosomal transmembrane protein, has emerged as a candidate regulator of these interconnected processes through its roles in lysosomal homeostasis, autophagic flux, receptor trafficking, metabolic adaptation, and therapeutic resistance.

**Main body:**

Experimental studies support important roles for LAPTM4B in lysosomal homeostasis, autophagic regulation, and multiple forms of therapy resistance. Additional evidence links autophagy-dependent stress adaptation to HIF-1α stabilization, glycolytic remodeling, lactate accumulation, and immunoregulatory changes within the TME. On the basis of these direct and indirect observations, this review proposes a conceptual model in which LAPTM4B may function as a context-dependent organizing node that helps couple lysosomal stress tolerance, metabolic adaptation, and immune escape in selected tumor settings. In this framework, LAPTM4B may contribute to sustained autophagic flux under stress, support metabolic conditions compatible with aerobic glycolysis, and potentially facilitate downstream immunosuppressive processes, including lactate-associated microenvironmental remodeling. We also review structural heterogeneity of LAPTM4B, clinical associations across multiple malignancies, multidrug-resistance mechanisms, and emerging evidence for EGFR-TKI escape mediated by LAPTM4B-dependent stabilization of ATP1A1 in non-small cell lung cancer.

**Conclusions:**

Current evidence supports LAPTM4B as an important regulator of lysosomal adaptation and therapy resistance, whereas its broader role in tumor immune evasion remains a testable model rather than an experimentally settled mechanism. We therefore present the autophagy-metabolism-immunity axis as a layered framework: experimentally supported in selected tumor contexts, indirectly supported by related metabolic and immune observations, and hypothesis-generating where direct causal evidence remains absent.

## 1 Introduction: from lysosomal homeostasis to microenvironment remodeling - a translational perspective

### 1.1 Lysosomes and autophagy: central hub for tumor stress and metabolic stability

Lysosomes are highly dynamic catabolic sites in cells and serve an essential critical role in the maintenance of physiological cellular homeostasis. Autophagy is an evolutionarily conserved lysosome-dependent degradation mechanism that captures and degrades damaged organelles, misfolded proteins and redundant macromolecules and delivers them to lysosomes for degradation and recycling of substances (1).

In the course of rapid solid tumor progression, tumor cells are often exposed to chronic and extreme microenvironmental stress, including hypoxia and severe depletion of amino acids and glucose; under such conditions, the autophagy pathway is generally highly upregulated and utilized by tumor cells, with the degradation products being re-released and recycled into metabolic and biosynthetic pathways to preserve mitochondrial metabolic function and global energy homeostasis, thereby conferring a significant survival advantage to tumor cells in the context of nutrient deprivation (1). Moreover, lysosome is not only a degradation site but also its surface acts as a central signaling hub integrating nutrient sensing signals (such as mTORC1 complex) and energy sensing (2). However, many studies have shown that the proper completion of autophagic flux is strictly regulated by the physicochemical homeostasis of lysosomes, particularly dependent on the maintenance of a steep acidic intraluminal pH gradient. If lysosomal homeostasis is disrupted, it will trigger apoptosis or significantly reduce the survival threshold of tumor cells to treatments (3). Thus, tight control of lysosomal homeostasis is an important strategy for tumor cells to survive metabolic stress and gain adaptive survival ability.

### 1.2 Structural characteristics, genetic polymorphisms, and functional differences of LAPTM4B isoforms

LAPTM4B is one of several membrane proteins implicated in the endomembrane system and lysosomal homeostasis. The LAPTM4B gene is located on human chromosome 8q22.1 and encodes a lysosome-associated protein with four hydrophobic transmembrane regions (4). Subsequent studies indicate that LAPTM4B can participate in endolysosomal signaling assemblies relevant to nutrient sensing and autophagy (5–7).

With respect to genetic polymorphisms, the 5’-untranslated region (5’-UTR) of the first exon of the LAPTM4B gene shows significant variations, mainly as allele *1 (containing a single 19-bp sequence) and allele *2 (containing two closely linked, tandem 19-bp repeat sequences) (8). Association studies have linked the LAPTM4B*2 allele to increased susceptibility or adverse clinicopathological features in selected cancers, including gastric and breast cancer cohorts (8, 9).

This gene primarily encodes two protein isoforms with substantial structural and functional differences: LAPTM4B-35 with a molecular weight of 35 kDa and LAPTM4B-24 with 24 kDa, which is caused by the use of alternative start codons (4). The LAPTM4B-24 isoform is expressed ubiquitously in normal healthy tissues; in the absence of the extreme N-terminal sequence, it has a rapid protein turnover rate (half-life of approximately 1 hour) and is regulated by nutrient abundance, mainly performing basic lysosomal nutrient-sensing functions (10). LAPTM4B-35 has an extra 91 amino acid N-terminal extension containing a proline-rich (PPRP) motif, and this isoform has been reported to be upregulated in multiple malignant tumor contexts (**Figure 1**) (11, 12). LAPTM4B-35 can interact with the small GTPase Cdc42 during cell migration and regulate integrin β1 recycling and focal adhesion dynamics, thereby promoting cancer cell migration and invasion (13). Its PPRP motif has also been linked to PI3K/AKT signaling activation and multidrug resistance (12, 14).

**Figure 1:**
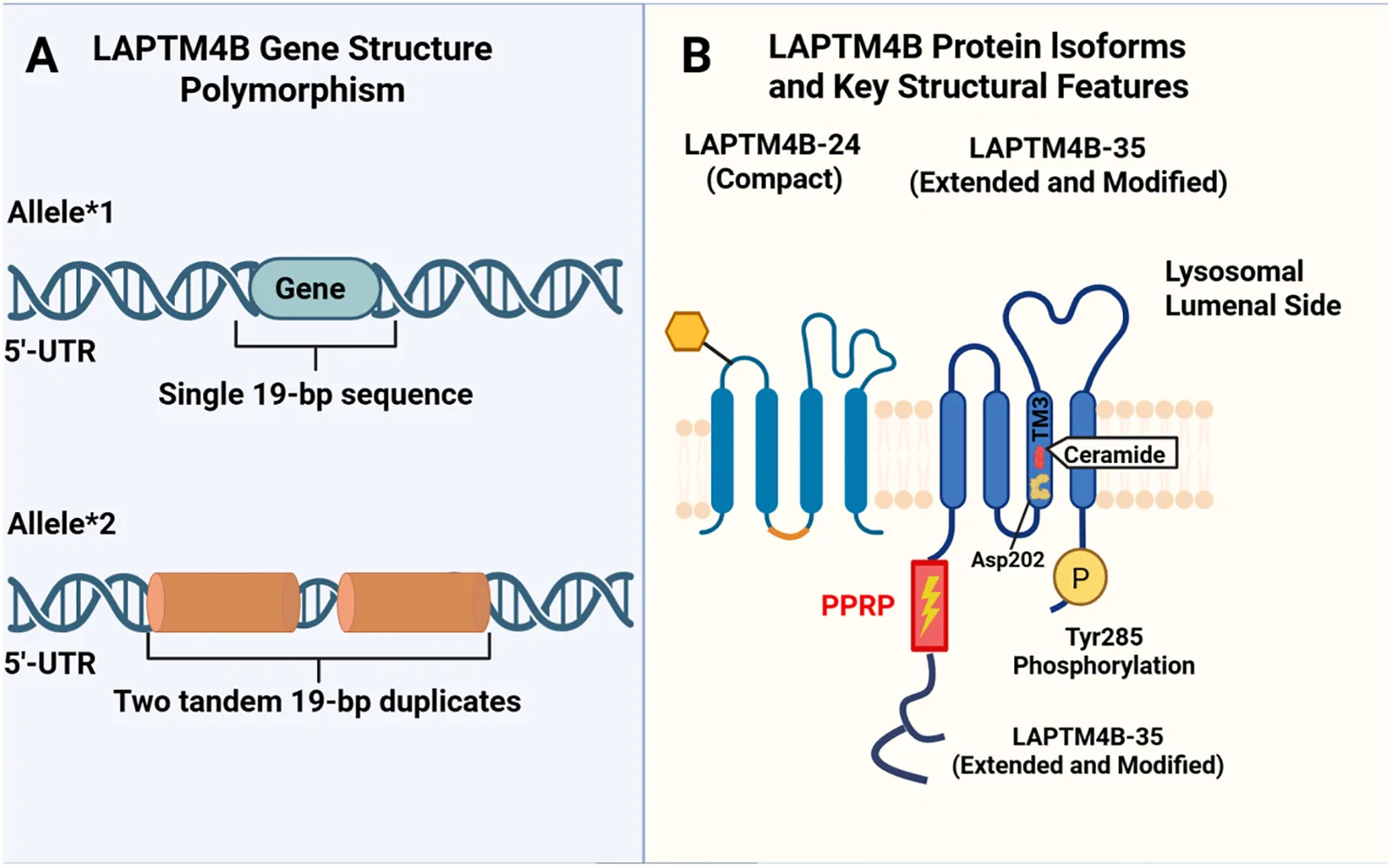
Genetic polymorphisms, structural isoforms, and core biochemical features of LAPTM4B. This schematic summarizes LAPTM4B heterogeneity at the genomic and protein levels. **(A)** The 5’ untranslated region of exon 1 contains a 19-bp sequence polymorphism. Allele *1 contains a single 19-bp segment, whereas allele *2 contains two tandem 19-bp repeats. LAPTM4B*2 has been associated with increased cancer susceptibility or poorer outcome in several tumor cohorts, although its clinical effect appears to vary by cancer type and population. **(B)** Alternative translation initiation generates two major isoforms. LAPTM4B-24 represents the shorter basal isoform, whereas LAPTM4B-35 contains an extended N-terminal cytoplasmic region with a proline-rich PPRP motif that can engage pro-survival signaling pathways such as PI3K/AKT. Additional structural elements, including a ceramide-sensitive region around the third transmembrane domain and C-terminal phosphorylation sites, support interactions with nutrient-sensing, lysosomal, and trafficking pathways. The figure therefore defines the structural basis for interpreting LAPTM4B as a context-dependent lysosomal and signaling adaptor rather than as a single invariant oncogenic driver across divergent tumor lineages. Created in BioRender. Haokun, L. (2026) https://BioRender.com/bap46hg.

### 1.3 Current research gaps and a proposed conceptual framework: a LAPTM4B-associated autophagy-metabolism-immunity axis

Although abundant basic and clinical literature supports an oncogenic role for LAPTM4B in tumor progression, many prior studies have focused on isolated signaling pathways (for example, EGFR or PI3K/AKT networks) (15) or have summarized LAPTM4B mainly through pan-cancer biomarker and immune-correlation analyses (16). Much less attention has been given to whether LAPTM4B-related lysosomal stress adaptation could be integrated with broader tumor ecological processes, including metabolic reprogramming and remodeling of the tumor immune microenvironment.

A recent LAPTM4B-focused review summarized the broader oncogenic functions and regulatory mechanisms of this protein across tumors (17). The present review is positioned differently: rather than providing another gene-centered catalogue, it uses an evidence-stratified framework to ask which LAPTM4B-related observations are directly demonstrated, which are indirectly supported, and which remain author-generated models linking autophagy, metabolism, and immune escape.

A conceptual basis for such integration comes from the framework proposed by Xia and colleagues in Nature Reviews Cancer, which emphasized reciprocal interactions among autophagy, metabolic reprogramming, and immune evasion during tumor progression. Building on that framework, this review distinguishes experimentally established findings from indirect supporting observations and then proposes a working model in which LAPTM4B may contribute to this higher-order network. Specifically, we suggest that under nutrient limitation and hypoxic stress, aberrantly elevated LAPTM4B may help preserve lysosomal function and autophagic flux, thereby creating conditions compatible with metabolic adaptation. We further propose that these effects may intersect with antigen-presentation defects and lactate-associated immunosuppression; however, these integrative links should be regarded as hypothesis-generating rather than as experimentally settled mechanisms.

Despite these advances, the immunological significance of LAPTM4B remains incompletely defined. For an immunology-focused readership, the central question is therefore not whether LAPTM4B is an oncogenic marker, but how strongly current evidence supports its placement upstream of antigen presentation, immune-cell recruitment, lactate-rich immunosuppression, and response to immune-checkpoint blockade.

## 2 Clinical relevance and multidimensional regulatory network of LAPTM4B across multiple malignancies

In HCC stem-like models, LAPTM4B has been shown to enhance CD133+ liver cancer stem-like cell stemness by activating WNT/β-catenin signaling and reducing β-catenin phosphorylation and ubiquitination-mediated degradation, partly through interactions with USP1 and USP14 (18). This supports a cancer-specific connection between LAPTM4B, stemness, and chemoresistance, while the functional necessity of these interactions and isoform-specific requirements should be validated across additional tumor types.

Recent large-scale multi-omics investigations suggest that LAPTM4B has broad diagnostic and prognostic relevance across several solid tumors and selected leukemias.

The distinct clinical phenotypes and multidimensional regulatory networks associated with LAPTM4B across diverse human malignancies, including gastrointestinal, respiratory, and hematological systems, are systematically summarized in **Supplementary Table 1**.

The evidence hierarchy used in this review is summarized in **Table 1**. Each mechanism is categorized according to the available experimental system, tumor context, and evidentiary strength, so that established findings are not conflated with indirect observations or proposed models.

**Table 1:** Evidence stratification for the LAPTM4B-centered framework.

Mechanistic module	Key LAPTM4B-related finding	Experimental system	Cancer context
Lysosomal homeostasis and autophagic flux	LAPTM4B supports lysosomal adaptation, late endosomal/lysosomal function, autophagosome-lysosome coupling, and stress survival.	Cell-line perturbation models, xenografts, and patient-expression associations.	HCC, breast cancer, lung cancer, glioma and other solid tumors.
mTORC1 and nutrient signaling	LAPTM4B has been linked to mTORC1-related growth signaling while also being associated with increased autophagic flux.	Mostly cell-based pathway assays and selected tumor models.	Primarily epithelial malignancies.
Upstream transcriptional control	HDAC2 can bind the LAPTM4B promoter, and a YAP-CREB1-LAPTM4B feedback loop may sustain HCC stemness.	Promoter assays, ChIP-related analyses, cell perturbation, xenograft/stemness assays.	Mainly HCC.
Stemness and Wnt/β-catenin	LAPTM4B-related signaling intersects with Wnt/β-catenin and stem-like phenotypes, including CD133+ liver cancer stem-like cells.	Cancer stem-like cell assays, pathway studies, and tumor models.	HCC and colorectal cancer.
MDSC recruitment and PD-L1 regulation	LAPTM4B can promote an ETV1-Wnt/β-catenin-CXCL8 axis that recruits MDSCs and affects PD-L1-related immune responses.	HCC cell models, immune co-culture/functional assays, animal models, and patient associations.	HCC.
MHC-I degradation hypothesis	LAPTM4B is proposed to contribute to antigen-presentation loss through NBR1-mediated MHC-I lysosomal degradation.	Indirect inference from lysosomal/autophagy biology and immune-evasion literature.	Hypothesis across solid tumors.
Lactate/lactylation axis	LAPTM4B may reinforce glycolysis and lactate-rich microenvironments that could support lactylation-mediated immune remodeling.	Bioinformatic and pathway-level inference plus lactate-related tumor-immunity evidence.	Proposed pan-cancer framework.
ccRCC immune phenotype	High LAPTM4B expression is associated with immune infiltration patterns including increased M2 macrophage abundance.	Patient-cohort bioinformatic and immune-infiltration analyses.	Clear-cell renal cell carcinoma.
Ph+ B-ALL progression	LAPTM4B deficiency suppresses B-ALL progression *in vivo* in knockout mouse models.	Genetic knockout mouse models and leukemia progression assays.	Ph+ B-ALL.
EGFR-TKI resistance	EGFR-TKI treatment can suppress WWP2-mediated LAPTM4B degradation, enabling LAPTM4B accumulation, ATP1A1 stabilization, and resistance.	EGFR-mutant lung-cancer cell and resistance models.	NSCLC.
Ferroptosis resistance	LAPTM4B promotes SLC7A11-related ferroptosis resistance, connecting lysosomal adaptation to redox therapy resistance.	Cell perturbation and pathway assays.	Tumor-type dependent.
sEV lipid remodeling	The SLim motif of LAPTM4B controls sphingolipid and ether-lipid signatures of small extracellular vesicles.	Biochemical and extracellular-vesicle lipidomic analyses.	Non-cancer and cancer-relevant cell systems.
miR-489-LAPTM4B-ICD axis	miR-489-induced suppression of LAPTM4B has been linked to immunogenic cell-death-like responses in breast cancer.	Breast-cancer cell models and immune-death marker assays.	Breast cancer.
Breast-cancer chemotherapy response	LAPTM4B copy-number gain has been associated with inferior response to anthracycline-based chemotherapy.	Clinical breast-cancer cohorts analyzed by FISH and treatment-response association.	Hormone receptor-negative breast cancer.
Multigene prognostic models	LAPTM4B appears in lysosome- or lactate-related multigene signatures associated with prognosis and immune features. Low-to-moderate correlative biomarker evidence; predictive utility requires prospective clinical validation.	Retrospective transcriptomic models and nomograms.	HCC and post-TACE HCC.

To improve conceptual clarity, the major components of the proposed LAPTM4B-centered network can be separated into mechanisms supported by direct experimental evidence, observations that provide indirect or associative support, and author-proposed integrative links that remain to be tested.

Across malignancies, LAPTM4B evidence is heterogeneous. HCC provides relatively strong mechanistic support for LAPTM4B-dependent Wnt/β-catenin signaling, stemness, CXCL8-mediated MDSC recruitment, and PD-L1-related phenotypes. NSCLC provides a clearer resistance mechanism involving EGFR-TKI-induced LAPTM4B stabilization. ccRCC currently offers mainly correlative immune-infiltration evidence, whereas Ph+ B-ALL provides direct *in vivo* genetic evidence for disease progression. These differences argue against presenting LAPTM4B as a uniformly proven pan-cancer immune-evasion driver; instead, they support a cancer-context-specific hub model.

## 3 Core driving chassis: control of lysosomal physicochemical homeostasis and spatial signaling by LAPTM4B

Several upstream mechanisms explain why LAPTM4B becomes persistently elevated in tumors. In HCC, HDAC2 has been reported to bind the LAPTM4B promoter and enhance LAPTM4B transcription (19). A second HCC study described a positive feedback loop in which LAPTM4B suppresses YAP ubiquitination, stabilized YAP cooperates with CREB1, and the YAP-CREB1 complex further promotes LAPTM4B transcription (20). These findings provide direct upstream regulatory mechanisms, but their current evidentiary scope is strongest in HCC and should not yet be generalized to all tumor types.

Before we discuss the high-dimensional ecological network of “autophagy-metabolism-immunity” in which the tumor cells gain microenvironmental remodeling, we need to clarify the chassis of physical space and chemical sensing on which it operates. In extreme stress environments with severe hypoxia and profound glucose and amino acid deprivation in the solid tumor core, the massively overexpressed LAPTM4B is by no means a passive downstream effector; rather, through its evolutionarily highly conserved four-transmembrane topology, it providesa critical molecular scaffold integrating autophagic flux and nutrient signaling at the endosome-lysosome interface.

LAPTM4B may help maintain lysosomal membrane integrity and reduce stress-associated lysosomal membrane permeabilization (LMP). Under metabolic and proteotoxic stress, this function may contribute to preservation of the acidic lysosomal gradient and limit cytoplasmic release of cathepsins such as cathepsin D (3). This lysosome-protective activity provides a biochemical basis by which tumor cells may sustain autophagic flux under stress.

In higher-order subcellular spatial coordination, LAPTM4B may build a non-kinase-dependent allosteric sensing and signaling distribution network. A specific lipid-binding motif on its third transmembrane helix (TM3) was found to acutely sense bioactive sphingolipids (especially ceramide) in late endosomes; this small transmembrane conformational rearrangement induced by lipid molecular binding specifically exposes binding sites on the cytosolic side, effectively recruiting the cell membrane amino acid transporter heavy chain 4F2hc (SLC3A2) complex to the lysosomal compartment (5). This cross-scale event of molecular network reorganization allows an inside-out activation mechanism of the vacuolar H+-ATPase (V-ATPase), thus transducing the physical acidic gradient signal in the lysosomal lumen to a spatial conformational change of the external Ragulator complex and the downstream activation signal of the mechanistic target of rapamycin complex 1 (mTORC1) (6). The method implies that LAPTM4B may convert the local metabolite concentration variation into global anabolic instructions.

Meanwhile, LAPTM4B can participate in regulating cellular signal transduction by mediating receptor-network reconfiguration during growth-factor limitation. In EGFR-related autophagy regulation, inactive EGFR can interact with LAPTM4B on endosomal membranes and recruit the Sec5-containing exocyst subcomplex (7). This interaction promotes dissociation of the autophagy inhibitor Rubicon from the Beclin 1 complex and supports basal and starvation-induced autophagy at the endosome-lysosome interface (**Figure 2**) (7).

**Figure 2:**
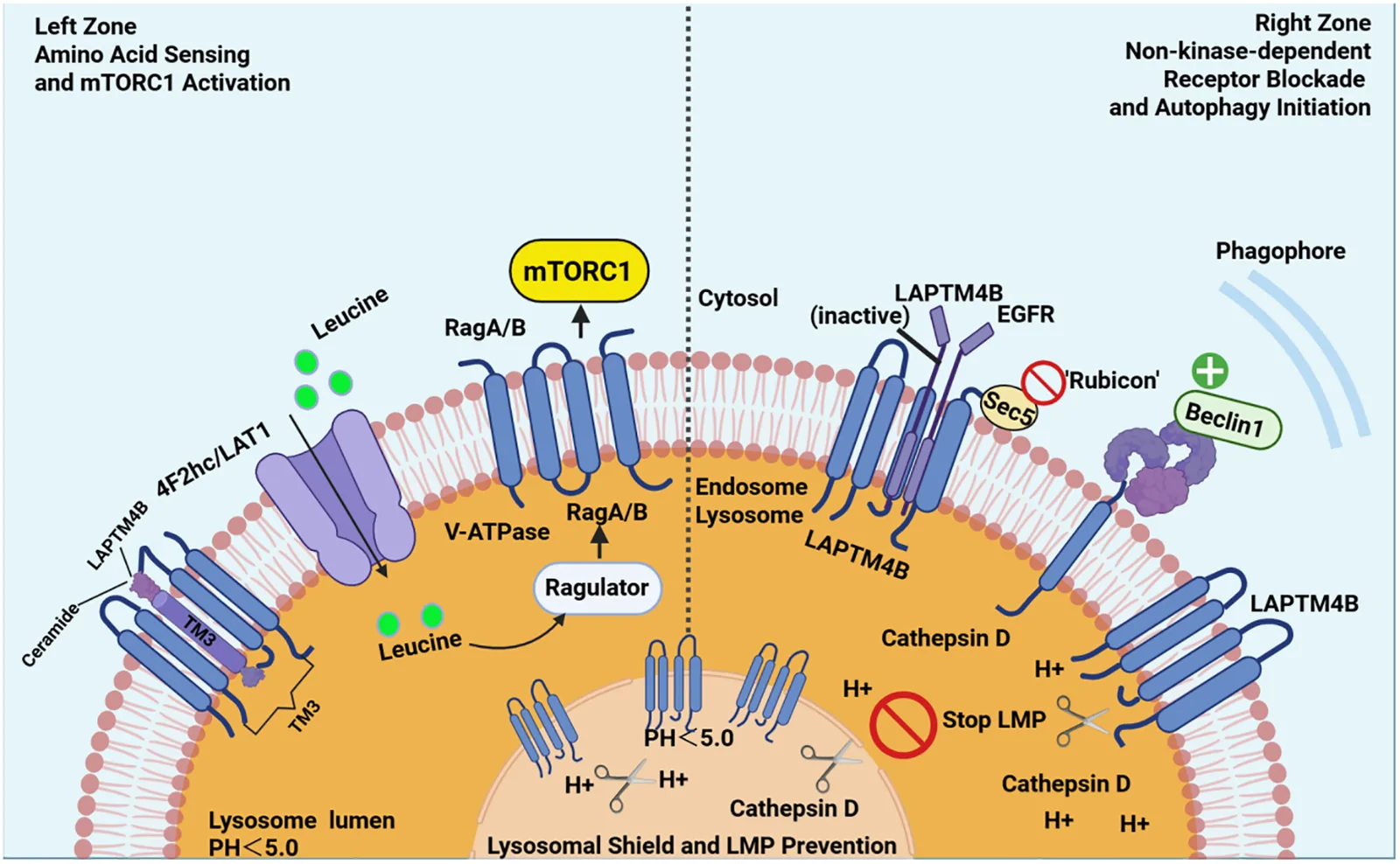
Context-dependent links between LAPTM4B, lysosomal signaling, mTORC1 activity, autophagy, and lysosomal membrane homeostasis. This model illustrates how LAPTM4B may organize several lysosome-centered processes through spatially distinct mechanisms. In the amino-acid sensing module, LAPTM4B has been reported to interact with the LAT1-4F2hc transporter complex and to support lysosomal leucine-dependent activation of Ragulator/Rag GTPase-mTORC1 signaling. In the receptor-trafficking module, LAPTM4B can bind endocytosed EGFR and cooperate with the exocyst component Sec5, promoting dissociation of Rubicon from the Beclin 1 complex and thereby facilitating autophagy initiation. In the lysosomal homeostasis module, elevated LAPTM4B may help preserve membrane integrity and limit lysosomal membrane permeabilization under stress, reducing cytosolic leakage of cathepsins and other hydrolytic enzymes. These modules should not be read as a single linear pathway. Instead, they highlight a potential spatial explanation for the apparent coexistence of mTORC1-related growth signaling and increased autophagic flux: LAPTM4B-associated effects may depend on compartment, timing, nutrient state, and treatment context. Created in BioRender. Haokun, L. (2026) https://BioRender.com/nhzmaj9.

In summary, LAPTM4B may systematically coordinate the preservation of physical membrane homeostasis (avoiding LMP-induced apoptosis), transmembrane transportation of chemical substances (uptake of vital amino acids, such as leucine, by LAT1/4F2hc), and the switch of cell survival signals (conformational dissociation of the Rubicon/Beclin 1 complex) at the same precise microscopic scale. The involvement of LAPTM4B could explain the biochemical basis for the later metabolic polarization of tumor cells towards the Warburg effect, the remodeling of the lactate microenvironment and the possible epigenetic immunosuppression imposed on the host immune system, providing continuous material flux and biological signal support.

The apparent coexistence of LAPTM4B-associated mTORC1 activation and enhanced autophagic flux should be interpreted as context-dependent rather than as a simple linear pathway. mTORC1 activation generally suppresses canonical autophagy initiation under nutrient-replete conditions, whereas hypoxia, lysosomal stress, treatment pressure, and spatially distinct lysosome pools can favor compensatory autophagic flux. Thus, LAPTM4B may support anabolic growth signaling in one temporal or subcellular context while preserving lysosomal-autophagic stress tolerance in another. Direct time-course and spatial lysosome studies are still required to resolve this issue.

## 4 An evidence-informed LAPTM4B-centric model of the “autophagy-metabolism-immunity” network

The reciprocal relationship among autophagy, metabolic reprogramming, and immune evasion is now well recognized in tumor biology. What remains less clear is whether specific lysosomal regulators help coordinate these processes under severe microenvironmental stress. Here, we outline an evidence-informed model in which LAPTM4B may serve such a coordinating function, while explicitly distinguishing direct experimental support from broader inference.

At present, available evidence most strongly supports roles for LAPTM4B in lysosomal homeostasis, autophagic regulation, growth-factor signaling, and therapy resistance. By contrast, its proposed position as an upstream coordinator linking autophagic flux to metabolic polarization, antigen-presentation defects, and lactate-driven immune remodeling remains inferential. We therefore discuss the following sections as a combination of established mechanisms, indirect support, and testable hypotheses.

A stepwise model is useful here. First, LAPTM4B alters lysosomal and endolysosomal function, which can preserve autophagic flux and membrane-protein trafficking. Second, these stress-adaptation programs interact with HIF-1α-associated glycolysis and lactate accumulation. Third, the altered metabolic and vesicular state can influence antigen presentation, immune-cell recruitment, macrophage polarization, and checkpoint responses. Feedback may occur because therapy, hypoxia, and immune pressure further stabilize or select LAPTM4B-high tumor cells.

### 4.1 Established links between autophagy control and metabolic reprogramming

Under hypoxic conditions and oncogenic stress, many tumor cells rely heavily on glycolysis and can produce large amounts of lactate even when oxygen is available, a metabolic phenotype commonly referred to as the Warburg effect (21). Independent studies also indicate that autophagy can support tumor survival during nutrient stress and may help sustain glycolytic adaptation in some settings (22, 23). Within this context, LAPTM4B is of interest because it has documented roles in lysosomal regulation and autophagic flux (3, 6, 7), suggesting that it may influence, rather than merely accompany, metabolic remodeling.

A plausible mechanistic intersection involves hypoxia-inducible factor-1α (HIF-1α), a key regulator of cellular responses to hypoxia and metabolic stress (24). Under normoxic conditions, HIF-1α is hydroxylated by prolyl hydroxylases (PHDs), recognized by the von Hippel-Lindau (VHL) E3 ligase complex, and targeted for proteasomal degradation (24, 25). Studies outside the specific LAPTM4B field show that mitochondrial reactive oxygen species and accumulation of TCA-cycle intermediates such as succinate can impair PHD activity and thereby stabilize HIF-1α (26–28). Because LAPTM4B supports stress-adaptive autophagy and endolysosomal homeostasis, it is reasonable to hypothesize that LAPTM4B-facilitated stress tolerance may help sustain intracellular conditions favorable for HIF-1α stabilization, although this causal chain has not yet been directly demonstrated.

If such a link is confirmed, stabilized HIF-1α would be expected to increase transcription of glycolysis-associated transporters and enzymes, including GLUT1, GLUT3, HK2, and LDHA (21, 24). Accordingly, the LAPTM4B-centered model should presently be viewed as a framework in which lysosomal adaptation may create permissive conditions for glycolytic reprogramming, rather than as definitive proof that LAPTM4B directly controls the full HIF-1α axis.

Several additional LAPTM4B-associated pathways could, in principle, reinforce this metabolic transition:

mTORC1-related nutrient signaling: LAPTM4B can recruit the LAT1-4F2hc amino acid transporter complex to lysosomes and promote mTORC1 activation (6). Because mTORC1 can enhance translation of growth-promoting programs and has been linked to HIF-1α/VEGF signaling, this pathway may cooperate with hypoxia-associated metabolic remodeling (29, 30).

PI3K/AKT-linked survival signaling: Through its N-terminal proline-rich motif, LAPTM4B can activate PI3K/AKT signaling (12, 14). This established survival pathway is compatible with enhanced anabolic growth and resistance to stress, and it may indirectly support glycolytic phenotypes in tumor cells.

Acid-export adaptation: High glycolytic flux requires effective management of lactate and proton accumulation. In some tumor models, autophagy-associated signaling has been linked to increased MCT1 expression through Wnt/β-catenin activation (23). These studies do not directly establish LAPTM4B as the driver of lactate export, but they support the broader idea that autophagy and metabolic waste handling can be functionally coupled.

Taken together, current evidence supports a connection between LAPTM4B, lysosomal stress adaptation, and pathways compatible with metabolic rewiring. The stronger claim that LAPTM4B functions as an upstream metabolic organizer should, however, be considered a testable interpretation rather than an established conclusion.

This metabolic interpretation should be bounded. The strongest support is that LAPTM4B is associated with HIF-1α-related glycolytic adaptation and treatment tolerance in selected cancer models. Whether LAPTM4B directly drives all downstream lactate-dependent immune phenotypes, however, remains uncertain and should be treated as indirect evidence unless LAPTM4B perturbation is coupled to metabolomic, lactylomic, and immune-functional readouts in the same system.

### 4.2 Evidence linking autophagy-mediated MHC-I degradation to a proposed role for LAPTM4B

Autophagy is often discussed as a stress-response pathway that supports tumor metabolism, but it can also influence immune recognition. This is particularly relevant when considering whether lysosomal regulators such as LAPTM4B might indirectly affect antigen presentation.

Importantly, direct evidence already shows that autophagy can mediate degradation of MHC-I through NBR1-dependent cargo trafficking in certain tumor contexts (31, 32). Separately, experimental studies support a role for LAPTM4B in autophagic flux, stress tolerance, and EGFR-linked autophagy regulation (3, 7). On the basis of these two observations, we propose that LAPTM4B may facilitate the intracellular environment required for efficient autophagy-mediated MHC-I turnover. However, direct experimental evidence placing LAPTM4B itself within the NBR1-MHC-I degradation pathway is currently lacking.

Accordingly, the LAPTM4B-MHC-I connection should be interpreted as a plausible extension of established autophagy biology rather than as a demonstrated mechanism. This distinction is important because the hypothesis remains attractive and testable, but it requires direct validation in models that simultaneously assess LAPTM4B expression, autophagic flux, MHC-I trafficking, and CD8+ T-cell recognition (**Figure 3**).

**Figure 3:**
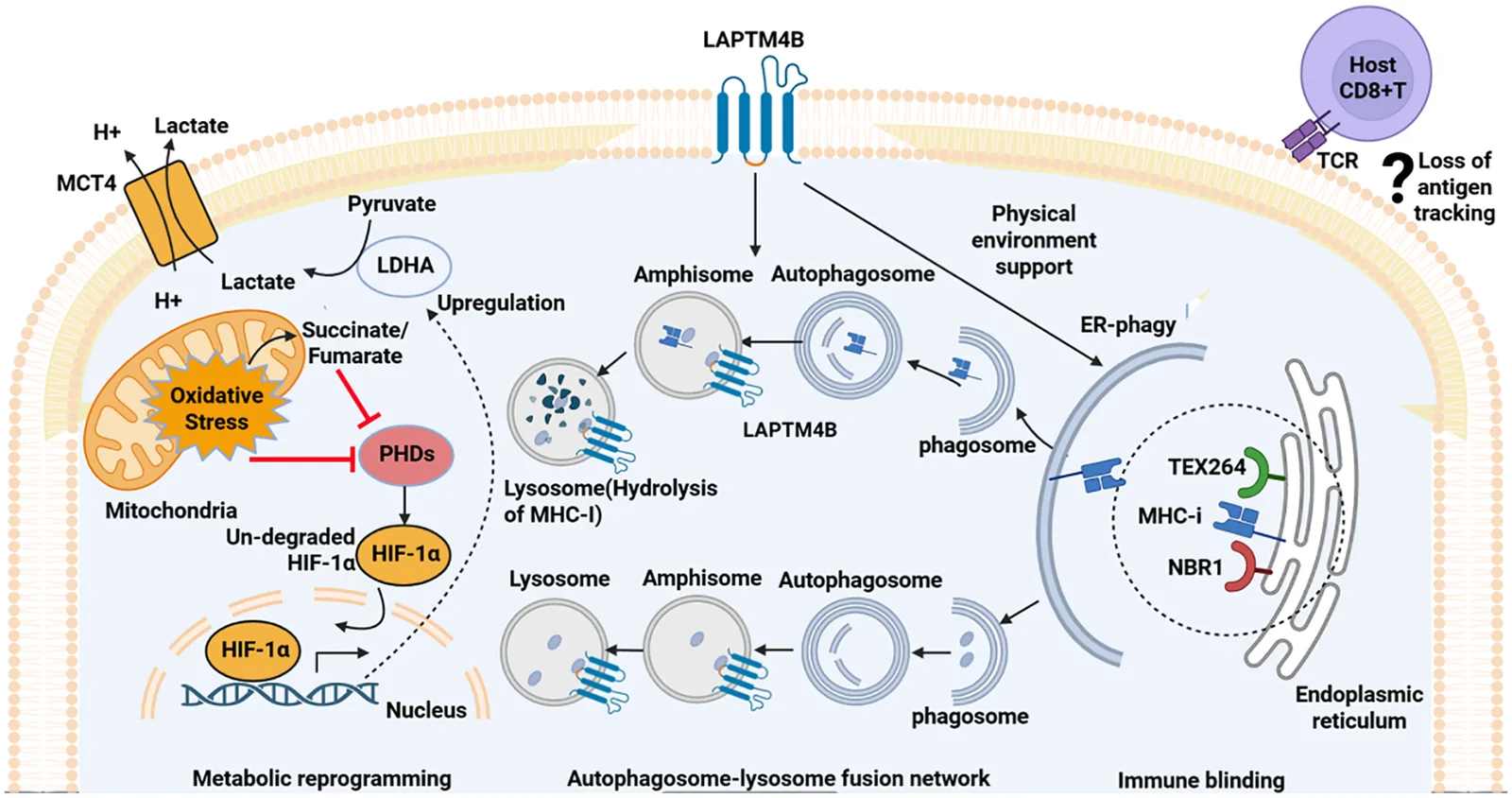
Proposed LAPTM4B-centered autophagy-metabolism-immunity network and its evidence boundaries. This schematic integrates established and hypothesis-generating links among autophagy, metabolic adaptation, and immune escape. At the center, LAPTM4B supports late endosomal/lysosomal adaptation and has been experimentally linked to autophagic flux and stress tolerance in cancer models. On the metabolic side, hypoxia, mitochondrial stress, and accumulated metabolites such as succinate or fumarate can stabilize HIF-1α by limiting prolyl hydroxylase activity. HIF-driven transcription of glycolytic and lactate-export programs, including LDHA and MCT transporters, may then promote extracellular acidification and lactate accumulation. On the immune side, autophagy-mediated MHC-I degradation through NBR1-dependent cargo trafficking has been demonstrated in selected tumor contexts. However, direct placement of LAPTM4B within the NBR1-MHC-I degradation pathway remains unproven. Therefore, the figure presents LAPTM4B as a plausible lysosomal enabling factor that may support conditions favorable for metabolic rewiring and immune evasion, while distinguishing this proposed connection from mechanisms that have already been directly validated. Created in BioRender. Haokun, L. (2026) https://BioRender.com/dkw8heg.

The NBR1-MHC-I mechanism should therefore be framed as a tissue-specific and testable extension rather than a universal conclusion. Direct evidence is still needed to show that LAPTM4B physically or functionally controls NBR1-dependent MHC-I sorting in different tumors, and that this mechanism changes CD8+ T-cell recognition *in vivo*.

### 4.3 Established lactate biology and a hypothetical LAPTM4B-centered lactylation axis

Lactate is no longer viewed merely as a metabolic by-product; it is now recognized as a signaling metabolite that shapes the biochemical and physical properties of the tumor microenvironment (33). High glycolytic activity can promote lactate accumulation and acidification of the extracellular tumor milieu (33–35).

A growing body of literature shows that lactate-rich, acidic environments influence stromal and immune cells and can contribute to histone lactylation and other regulatory responses (36, 37). These findings are established at the level of tumor metabolism and microenvironmental signaling, even though they do not by themselves identify LAPTM4B as the upstream controller of the entire process.

Mechanistically, aminoacyl-tRNA synthetases such as AARS1 and AARS2 have been reported to act as lactate sensors and lysine lactyltransferases in lactate-responsive signaling networks (38).

Lactate-associated chromatin remodeling has also been linked to increased histone lactylation at sites such as H3K18la, H3K9la, and H3K14la, with downstream effects on transcriptional accessibility and gene regulation (36).

Beyond histones, lactate-responsive regulation may extend to non-histone targets, including METTL3-associated RNA modification networks and cGAS-related immune signaling (38, 39).

Within the framework proposed here, LAPTM4B is not presented as a proven direct inducer of all lactylation events. Rather, we suggest that if LAPTM4B helps sustain autophagic and metabolic conditions favoring high lactate output, it could indirectly contribute to a lactate-rich microenvironment that promotes these downstream epigenetic and immunoregulatory changes.

Accordingly, LAPTM4B-linked lactylation should be described as a plausible metabolic-epigenetic bridge. The necessary evidence would include LAPTM4B gain- and loss-of-function studies, isotope tracing of lactate production, global lysine lactylome profiling, and rescue experiments connecting specific lactylated targets to immune-cell recruitment or T-cell dysfunction.

### 4.4 A proposed immunosuppressive output of the lactate/lactylation axis

Based on the metabolic and epigenetic observations summarized above, it is reasonable to hypothesize that a LAPTM4B-associated network could contribute to an immunosuppressive microenvironment. Here again, the downstream biology of lactate and immune dysfunction is better established than the more specific claim that LAPTM4B is the singular upstream coordinator.

Dimension 1: Recruitment of suppressive myeloid populations. Pan-cancer immune-correlation analysis and HCC-focused mechanistic work have linked high LAPTM4B expression to immune-remodeled tumor contexts (16, 40). In hepatocellular carcinoma, LAPTM4B-associated signaling has been connected to CXCL8 production and MDSC recruitment (40). These findings support an association between LAPTM4B-related programs and suppressive myeloid infiltration, although the extent to which this generalizes across tumor contexts remains to be defined.

Mechanistically, HCC data support an ETV1-Wnt/β-catenin-CXCL8 cascade in which LAPTM4B contributes to MDSC recruitment and PD-L1-associated immune regulation (40). Importantly, this does not mean that LAPTM4B-high tumors are uniformly resistant to immunotherapy. In this context, LAPTM4B-driven PD-L1 regulation may also increase tumor dependence on the PD-1/PD-L1 axis, potentially enhancing sensitivity to PD-L1 blockade under selected conditions. Therefore, LAPTM4B should be interpreted as a context-dependent immune-regulatory factor rather than a unidirectional marker of immunotherapy resistance.

Dimension 2: Treg-favoring metabolic conditions. Independent studies show that Tregs remain comparatively adaptable in high-lactate, low-glucose environments, whereas conventional effector T cells often become metabolically compromised (41). Additional work suggests that lactylation-related mechanisms, including MOESIN-associated TGF-β signaling and other lactylation-associated regulatory changes, may support suppressive immune states (42, 43). Within our model, these observations represent downstream consequences that could be amplified if LAPTM4B contributes to persistent lactate-rich conditions, but this indirect connection has not yet been directly resolved.

Dimension 3: Metabolic suppression of cytotoxic lymphocytes. Elevated tumor-derived lactate has been shown to blunt T-cell and NK-cell tumor immunosurveillance, while independent work indicates that NK-cell antitumor activity is strongly shaped by metabolic programs such as NAD+ salvage-dependent mitochondrial function (34, 44). Studies on FIP200 regulation further support the idea that lactate-associated metabolic stress can undermine survival of effector T cells (45). These data strongly support lactate-associated immune dysfunction, while the specific placement of LAPTM4B at the top of this axis remains a proposed interpretation.

Additional immune evidence is emerging outside HCC. In clear-cell renal cell carcinoma, LAPTM4B-high tumors have been associated with altered immune infiltration, including increased M2 macrophage abundance (46). In Ph+ B-ALL, pan-cancer and leukemia-focused analyses supported an oncogenic role for LAPTM4B, and genetic LAPTM4B deficiency suppressed leukemia progression *in vivo* (16). These studies strengthen the immunological relevance of LAPTM4B, but they differ in evidence type: ccRCC remains largely correlative, whereas the B-ALL data include direct animal-model evidence.

### 4.5 Proposed feedback loops and spatial metabolic symbiosis

If LAPTM4B does participate in this broader network, then one prediction is that the system would contain reinforcing feedback loops and spatially organized metabolic cooperation. The following features are therefore presented as conceptual extensions built on published findings rather than as directly proven properties of LAPTM4Bitself.

Endogenous “metabolism-epigenetics-autophagy” feedback loop: Lactate can act not only on immune cells but also back on tumor cells. In colorectal cancer models, lactate uptake has been linked to H3K18la remodeling and transcriptional upregulation of RUBCNL (Pacer), which supports autophagosome maturation (47). These observations provide a plausible mechanism by which metabolic output could feed back into autophagy. We propose that LAPTM4B may strengthen the conditions under which such a loop becomes sustained, but direct evidence for a LAPTM4B-RUBCNL circuit is not yet available.

Single-cell transcriptomic evidence has further identified a LAPTM4B-positive stem-like colorectal cancer subgroup that appears partly independent of the classical LGR5-positive compartment and is associated with poor prognosis (48). This finding strengthens the clinical relevance of LAPTM4B in colorectal cancer, but it should be interpreted as phenotype-level and tumor-specific evidence rather than direct proof of the full autophagy-metabolism-immunity model.

Spatial metabolic symbiosis: Peripheral tumor cells with better oxygenation can import lactate released by hypoxic core cells through MCT1 and use it as a mitochondrial carbon source (49). This type of metabolic compartmentalization is experimentally supported in tumor ecology. In the present model, we suggest that LAPTM4B-dependent stress adaptation may be compatible with, and perhaps supportive of, this spatial organization, while acknowledging that this relationship remains inferential.

Lineage heterogeneity and parallel stress-resistance programs: LAPTM4B-associated immune phenotypes are unlikely to be identical across all cancers. Indeed, pan-cancer analyses suggest contextual differences, including distinct associations in central nervous system tumors (16). In addition, LAPTM4B can stabilize SLC7A11 and suppress ferroptosis in non-small cell lung cancer (50), indicating that ferroptosis resistance may represent a parallel survival arm in some genetic backgrounds. We therefore view immune evasion, metabolic plasticity, and ferroptosis resistance as context-dependent branches of a broader hypothesis rather than a single uniform pathway.

Lipid metabolic compensation and extracellular-vesicle communication: LAPTM4B can sense ceramide-related membrane stress and influence multivesicular body trafficking and ceramide-induced exosome release (5, 51). More broadly, exosome studies support a role for extracellular vesicles in intercellular communication and pre-metastatic niche formation (52). These data support the possibility that LAPTM4B contributes to adaptive vesicular communication under therapeutic or metabolic pressure, although a comprehensive systems-level model remains to be experimentally assembled. These proposed relationships are summarized in **Figure 4**.

**Figure 4:**
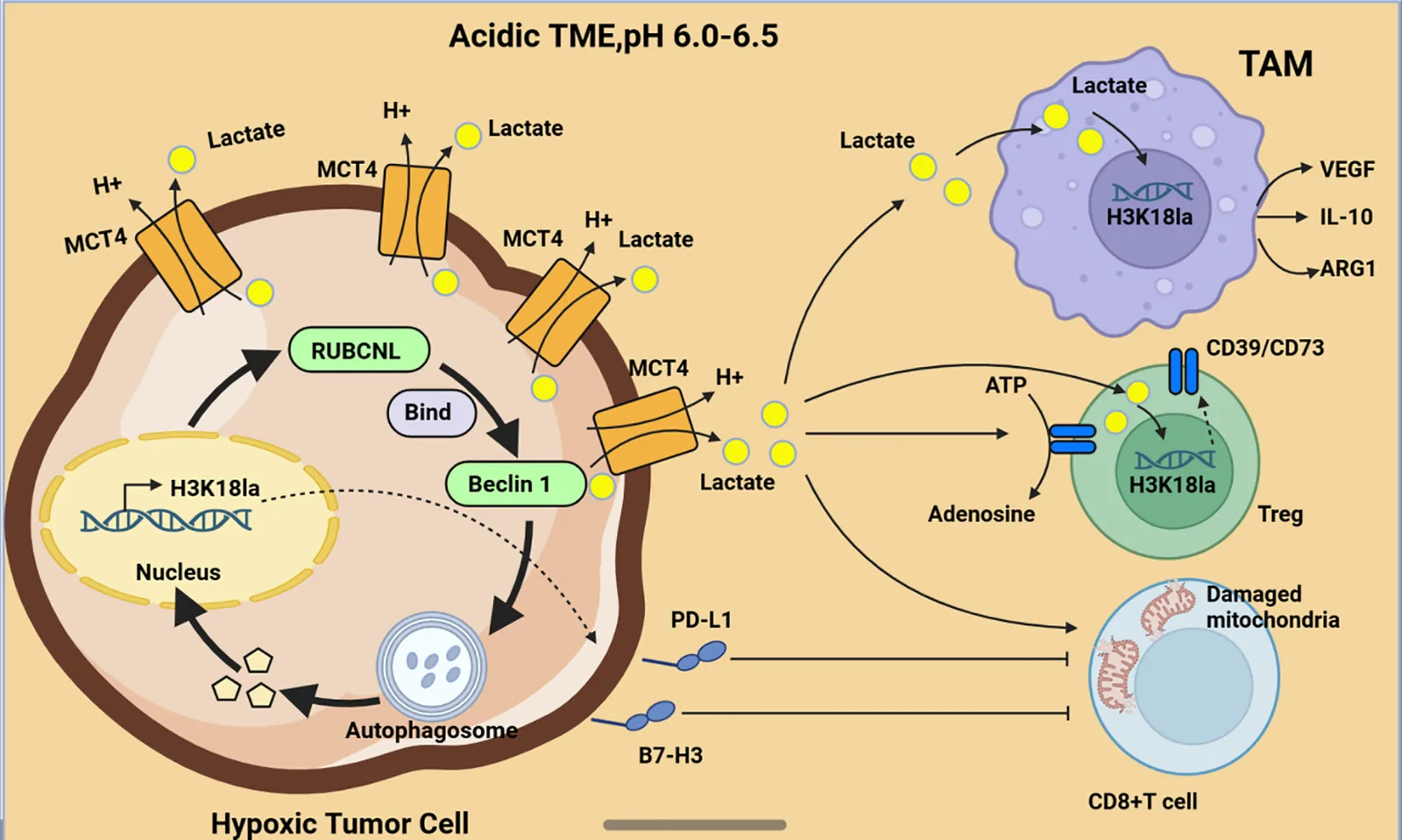
Lactate-associated immunometabolic remodeling and proposed feedback with autophagy-related tumor adaptation. This model depicts how lactate-rich tumor microenvironments may reinforce immune suppression and stress adaptation. Hypoxic or metabolically rewired tumor cells can export lactate and protons through transporters such as MCT4, generating an acidic extracellular niche. Lactate can function not only as a metabolic byproduct but also as a signaling and epigenetic substrate, including through histone lactylation and related regulatory mechanisms. These processes have been associated with macrophage polarization, regulatory T-cell adaptation, adenosine-generating ectonucleotidases, and impaired cytotoxic T-cell or NK-cell activity. In some settings, lactate-related signaling may also influence immune-checkpoint expression, but these effects are context dependent and should be interpreted according to tumor lineage and experimental setting. The left side illustrates a proposed feedback model in which lactate-associated epigenetic remodeling could enhance autophagy-supporting programs such as RUBCNL-Beclin 1 signaling, thereby sustaining survival under stress. This feedback loop remains a hypothesis-generating extension unless experimentally connected to LAPTM4B in specific tumor systems. Created in BioRender. Haokun, L. (2026) https://BioRender.com/awz1x53.

LAPTM4B may also influence intercellular communication through small extracellular vesicles. The SLim motif of LAPTM4B has been shown to regulate the sphingolipid and ether-lipid signature of small extracellular vesicles (53). This expands LAPTM4B biology beyond lysosomal stress adaptation, but the immune consequences of LAPTM4B-dependent vesicle lipid remodeling in tumors remain to be experimentally defined.

## 5 Translational clinical applications of LAPTM4B in tumor targeted therapeutics and precision diagnostics: from molecular biomarkers to precision targeted intervention networks

### 5.1 From basic mechanisms to multimodal precision molecular monitoring and translation to medicinE

In precision oncology, LAPTM4B has evolved from being viewed solely as a pro-proliferative oncogene to being considered a candidate integrator of lysosomalstress adaptation, therapy resistance, and tumor microenvironment remodeling in selected disease contexts.

In the setting of liquid biopsy and dynamic monitoring, circulating LAPTM4B may have potential as a biomarker of tumor burden and treatment response. Serum LAPTM4B changes have been reported to track adjuvant therapy response in breast cancer (54), serum LAPTM4B-35 has been evaluated as a diagnostic marker in hepatocellular carcinoma (55), and serum LAPTM4B-35 combined with CA19–9 improves early detection performance in pancreatic ductal adenocarcinoma (56). More broadly, exosome biology may reflect systemic communication states linked to micrometastatic risk and pre-metastatic niche formation (52), while magnetic nanoparticle chemiluminescence enzyme immunoassay (MPs-CLEIA)-based assays support sensitive detection in urine and may have value in urological tumor screening (57).

For circulating tumor cells (CTCs), peptide biomimetic nano-interfaces targeting extracellular regions of LAPTM4B may complement EpCAM-based approaches by enriching invasive EMT-like populations for downstream single-cell analysis (58). Likewise, 18F-labeled PET probes and near-infrared imaging probes directed against LAPTM4B indicate potential opportunities for non-invasive lesion tracking and image-guided surgery (59, 60). These applications remain promising but will require broader clinical validation.

### 5.2 Overcoming physical and molecular barriers to reconstruct anti-tumor drug resistance mechanisms

Drug resistance remains a major clinical manifestation of LAPTM4B biology, but the evidentiary weight differs across examples. In hormone receptor-negative breast carcinomas, LAPTM4B gene copy-number gain has been associated with inferior response to anthracycline-based chemotherapy (61). Stronger current mechanistic examples include the EGFR-TKI-WWP2-LAPTM4B-ATP1A1 loop in NSCLC (62) and SLC7A11-related ferroptosis resistance (50). In addition, LAPTM4B expression has been associated with adverse prognosis in triple-negative breast cancer (63).

In targeted therapy, recent work suggests that LAPTM4B accumulation under sustained EGFR-TKI pressure can stabilize ATP1A1, alter vesicular acidification, and delay degradation of activated EGFR in non-small cell lung cancer models (62). These findings provide a mechanistically specific example of how LAPTM4B-associated endomembrane regulation may contribute to acquired resistance and may help explain why LAPTM4B suppression enhances EGFR-TKI sensitivity in experimental systems.

The upstream part of this resistance loop is important. EGFR-TKI exposure can reduce WWP2-mediated LAPTM4B degradation, leading to LAPTM4B accumulation. Increased LAPTM4B then stabilizes ATP1A1, reinforcing survival signaling and further reducing EGFR-TKI sensitivity (62). This creates a positive feedback model: TKI treatment→ impaired LAPTM4B degradation→ LAPTM4B accumulation→ATP1A1 stabilization→ persistent drug resistance.

LAPTM4B-associated stress protection may also extend to radiotherapy and ferroptosis-related resistance. In head and neck cancer models, radiation-enhanced LAPTM4B-EGFR-Beclin1 signaling has been linked to protective autophagic flux (7, 64). Separately, LAPTM4B-mediated stabilization of SLC7A11 can preserve antioxidant capacity and reduce ferroptotic vulnerability (50). Together, these findings support the view that LAPTM4B contributes to parallel resistance programs in at least some tumor contexts.

### 5.3 Microenvironmental remodeling and stratification of immunotherapy sensitivity

The efficacy of immune checkpoint inhibitors is strongly influenced by the metabolic state of the tumor microenvironment. Because LAPTM4B is associated with autophagic stress adaptation, glycolytic signaling, and immune-remodeled niches, it is reasonable to explore whether LAPTM4B-related signatures may help identify tumors prone to metabolically mediated immunotherapy resistance. Experimental evidence already supports lactate-driven immunosuppression and MDSC-rich states in several cancers; the more specific LAPTM4B-centered interpretation remains an important but incompletely resolved translational question.

Accordingly, future clinical models might integrate circulating LAPTM4B, systemic lactate-related measurements, and spatial immune features such as MDSC infiltration to identify patients at high risk for poor response to immune checkpoint blockade. This should presently be viewed as a translational hypothesis that merits prospective validation rather than as a ready-to-implement clinical algorithm.

For immunotherapy, the direction of effect should be presented carefully. LAPTM4B can support immune-suppressive features such as MDSC recruitment and PD-L1 regulation in HCC (40), yet increased PD-L1 dependence may also create sensitivity to PD-L1 blockade in selected models. Therefore, LAPTM4B should be evaluated as a context-dependent biomarker of immune remodeling rather than as a simple marker of universal checkpoint resistance.

### 5.4 Future paradigms and precision degradation strategies for multidimensional intervention networks

If LAPTM4B proves to be a functionally important node in therapy resistance and tumor adaptation, then it may become an attractive intervention target. At the same time, LAPTM4B remains challenging from a drug-discovery perspective because it is a four-pass transmembrane protein lacking a conventional kinase-like binding pocket.

One route is small-molecule antagonism. Ethoxalyl thiosemicarbazone (ETS) has been reported to interact with LAPTM4B-35, interfere with Tyr285 phosphorylation and LAPTM4B-PI3K p85α interaction, and suppress downstream PI3K/AKT signaling (**Figure 5B**) (65). Although these findings are still early, they support the feasibility of developing allosteric or interface-disrupting strategies against LAPTM4B-related functions.

**Figure 5:**
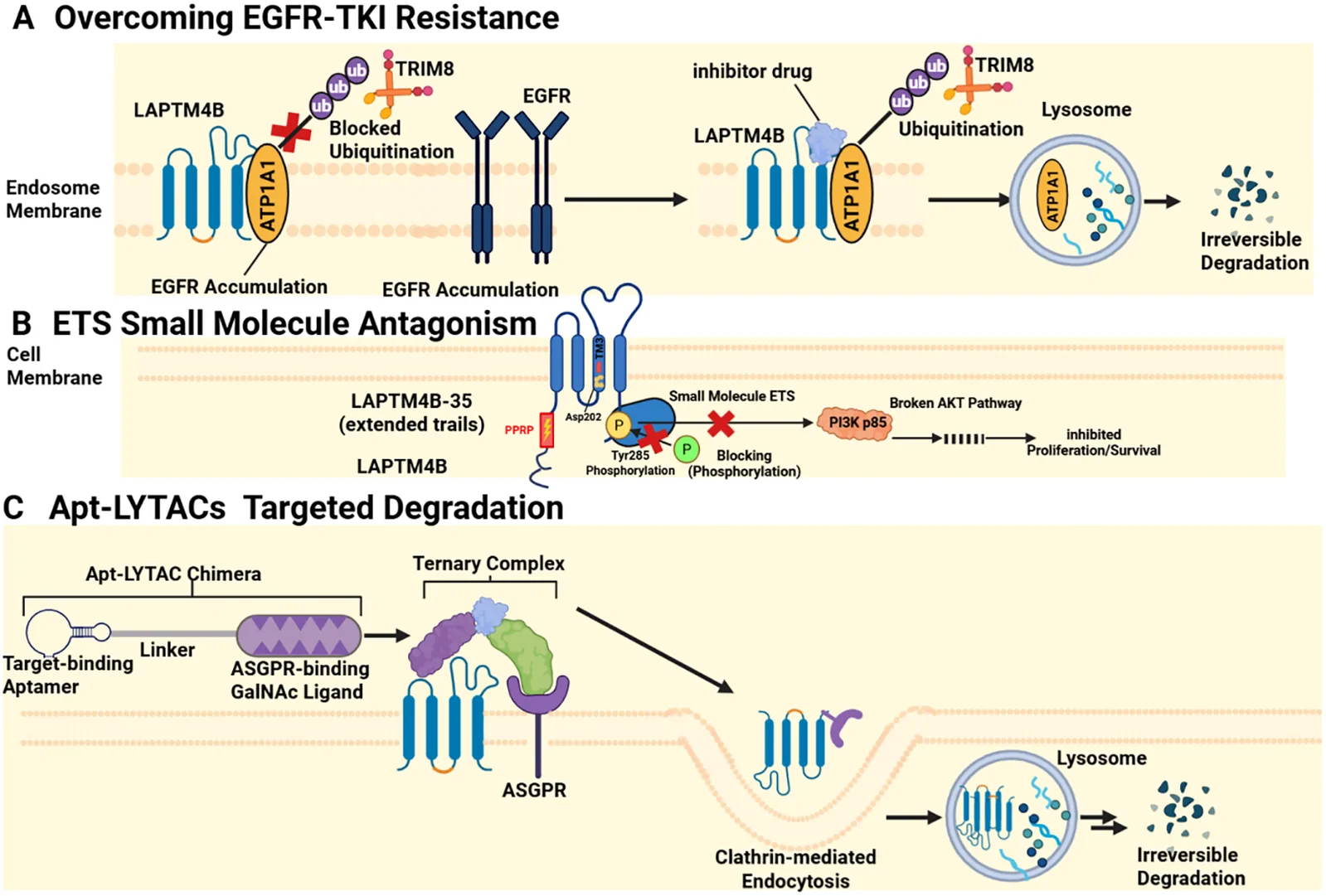
Translational opportunities for targeting LAPTM4B-associated resistance and lysosomal adaptation. This figure summarizes potential therapeutic strategies emerging from LAPTM4B biology. **(A)** In EGFR-TKI-treated non-small cell lung cancer models, sustained drug pressure can reduce WWP2-mediated LAPTM4B degradation, leading to LAPTM4B accumulation. Increased LAPTM4B stabilizes ATP1A1, alters vesicular acidification, delays degradation of activated EGFR, and contributes to acquired EGFR-TKI resistance. Therapeutic suppression of this loop may therefore restore drug sensitivity, although clinically validated LAPTM4B-directed inhibitors are not yet available. **(B)** Small-molecule antagonism is represented by ETS, which has been reported to interfere with LAPTM4B-35-associated signaling, including PI3K/AKT pathway activation. This remains a preclinical strategy requiring further optimization and validation. **(C)** Targeted degradation approaches, including LYTAC or aptamer-LYTAC concepts, could in principle redirect extracellular or membrane-accessible LAPTM4B toward lysosomal clearance. These approaches are conceptually attractive because LAPTM4B is a multi-pass membrane protein, but target accessibility, isoform specificity, delivery, safety, and tumor-type selectivity remain unresolved translational challenges. Created in BioRender. Haokun, (L) (2026) https://BioRender.com/dhxq324.

A second route is targeted protein degradation or post-transcriptional suppression. Lysosome-targeting chimeras (LYTACs) and aptamer-LYTAC platforms offer a conceptual means of directing extracellular or membrane proteins toward lysosomal clearance (66). In principle, LAPTM4B-directed degraders could be explored in the future, while RNA-based strategies such as miR-188-5p-mediated repression of LAPTM4B or broader miRNA approaches affecting autophagy and chemoresistance provide complementary suppression concepts (67, 68). At present, these approaches should be viewed as promising translational concepts rather than clinically mature LAPTM4B-directed interventions.

A second RNA-based axis has recently connected miR-489 to LAPTM4B inhibition and immunogenic-cell-death-like responses in breast cancer (69). This finding supports the idea that suppressing LAPTM4B may alter tumor-cell immunogenicity, but it remains an emerging mechanism that requires immune-competent *in vivo* validation.

A third route is to target network outputs rather than LAPTM4B directly. Inhibitors of lactate transport, such as the MCT1-directed agent AZD3965, may help counteract lactate-dependent metabolic vulnerabilities in selected tumor settings (70). More broadly, elevated lactate-related metabolic states are associated with diminished antitumor immunity and poorer responses to immune checkpoint blockade, supporting the rationale for testing metabolic reconditioning strategies where LAPTM4B-related programs coexist with lactate-driven immune dysfunction (35, 40).

Overall, the translational landscape around LAPTM4B spans biomarker development, resistance reversal, and targeted degradation concepts. **Supplementary Table 2** summarizes this translational roadmap, including circulating and imaging biomarkers, resistance-associated mechanisms, small-molecule antagonism, RNA-based suppression, LYTAC-related degradation concepts, lactate-transport targeting, and rational combination strategies (3, 14, 35, 40, 50, 52, 54–60, 62, 64–68, 70, 71). These opportunities are promising, but their prioritization should be guided by direct mechanistic validation of which parts of the LAPTM4B-centered framework are broadly operative versus tumor-type specific.

Clinically, LAPTM4B also appears in multigene predictive models rather than acting only as a single-gene marker. A lysosome-related three-gene nomogram including LAPTM4B, PRKCD, and LPCAT1 showed prognostic and therapy-prediction value in HCC (72). A lactate-related post-TACE HCC signature including LAPTM4B was associated with immunosuppressive features and poor outcome (73). These findings are useful correlative biomarkers, but they should be considered lower-level evidence until prospectively validated.

### 5.5 Testable predictions of the LAPTM4B-centered model

A useful feature of a conceptual review is that it should generate experimentally tractable predictions. If LAPTM4B is genuinely more than a biomarker or local signaling regulator, then perturbing LAPTM4B should alter not only lysosomal stress adaptation but also selected downstream metabolic and immune outputs. Several testable predictions emerge from the framework proposed here.

First, direct perturbation of LAPTM4B should affect MHC-I trafficking or degradation under conditions in which autophagy-mediated antigen-presentation loss is observed. This prediction could be examined by combining LAPTM4B knockdown or overexpression with assays of autophagic flux, MHC-I surface abundance, intracellular trafficking, and CD8+ T-cell recognition.

Second, if LAPTM4B contributes functionally to immunometabolic rewiring, then its inhibition should measurably alter lactate output, extracellular acidification, or lactylation-associated chromatin signatures under defined stress conditions. Such studies would help distinguish whether LAPTM4B is upstream of these events or instead reflects a parallel adaptation marker.

Third, LAPTM4B-directed perturbation should reshape the immune consequences of tumor stress adaptation in model systems that preserve microenvironmental context. Relevant readouts could include MDSC recruitment, Treg-associated signatures, NK-cell activity, and CD8+ T-cell fitness in co-culture, organoid, or *in vivo* settings.

Fourth, the proposed network is unlikely to operate uniformly across all cancers. Comparative studies across tumor lineages, isoform states, and treatment contexts should therefore determine whether the LAPTM4B-centered model reflects a broadly conserved adaptive program or a set of context-dependent branches with distinct translational implications.

Fifth, isoform- and dose-specific experiments are needed. Future studies should compare LAPTM4B-24 and LAPTM4B-35 rescue constructs, graded expression systems, organoids, immune-competent or humanized mouse models, and spatial profiling of immune neighborhoods. This would define whether LAPTM4B mechanisms are driven by expression level, isoform-specific motifs, tumor stage, or tissue-specific lysosomal programs.

## 6 Limitations, evidence boundaries, and future directions

### 6.1 Limitations of current evidence

Current LAPTM4B evidence has several module-specific limitations. First, many autophagy studies rely on cell lines and endpoint marker changes rather than dynamic flux assays in immune-competent tumors. Second, metabolic studies often infer HIF-1α-glycolysis-lactate relationships without integrating metabolomics, isotope tracing, and immune-functional assays in the same models. Third, immune-evasion mechanisms are uneven: HCC provides direct evidence for CXCL8/MDSC/PD-L1 regulation, whereas MHC-I degradation, lactylation-driven immune remodeling, and pan-cancer T-cell suppression remain largely hypothesis-generating. Fourth, clinical evidence is often retrospective and transcriptomic, with limited prospective validation. Finally, isoform-specific roles, polymorphism effects, dose dependence, tumor-stage dependence, organoid models, and humanized mouse studies remain insufficient.

### 6.2 Practical directions for validation

Future work should move from broad association to mechanism-matched validation. For basic biology, studies should combine LAPTM4B gain/loss/rescue experiments with autophagic flux, lysosomal positioning, mTORC1 activity, metabolomics, lactylomics, antigen-presentation assays, and immune killing assays. For translational validation, large clinical cohorts should test LAPTM4B isoforms, copy-number alteration, promoter regulation, and protein localization alongside immune infiltration and treatment response. For drug development, LAPTM4B-targeted inhibitors, degraders, antibodies, or RNA-based strategies should be evaluated with pharmacodynamic biomarkers and rational combinations, including EGFR-TKIs, ferroptosis-inducing therapy, chemotherapy, and immune-checkpoint blockade.

## 7 Discussion and conclusions

Taken together, LAPTM4B is best viewed as a context-dependent lysosomal adaptation factor with important consequences for tumor survival, therapy resistance, and immune remodeling. The most secure evidence supports roles in lysosomal/endolysosomal biology, autophagy-associated stress tolerance, oncogenic signaling, and selected resistance mechanisms. Strong cancer-specific immune evidence is emerging in HCC and Ph+ B-ALL, whereas ccRCC and multigene biomarker studies currently provide mainly correlative support. The broader autophagy-metabolism-immunity axis remains a useful organizing model, but not every link is experimentally proven. A balanced interpretation is therefore that LAPTM4B may coordinate lysosomal, metabolic, and immune programs in selected tumors, while its pan-cancer centrality requires systematic validation across tumor type, stage, isoform, and treatment context.

## Glossary

5’-UTR: 5’-untranslated region
AARS: aminoacyl-tRNA synthetase
AREs: adenine-uridine-rich regions
ASGPR: asialoglycoprotein receptor
AT: adjuvant therapy
Cgas: cyclic GMP-AMP synthase
CSCs: cancer stem cells/stem cell-like subpopulations
CTCs: circulating tumor cells
DAMPs: damage-associated molecular patterns
EGFR: epidermal growth factor receptor
EMT: epithelial-mesenchymal transition
ETS: ethoxalyl thiosemicarbazone
GBM: glioblastoma multiforme
GLUT: glucose transporter
HCC: hepatocellular carcinoma
HIF-1α: hypoxia-inducible factor 1 alpha
HK2: hexokinase 2
ICB: immune checkpoint blockade
ICIs: immune checkpoint inhibitors
Kla: histone lactylation
LAPTM4B: lysosomal-associated protein transmembrane 4 beta
LAT1: L-type amino acid transporter 1
LDHA: lactate dehydrogenase A
LMP: lysosomal membrane permeabilization
LNPs: lipid nanoparticles
LYTACs: lysosome-targeting chimeras
MCT: monocarboxylate transporter
MDSCs: myeloid-derived suppressor cells
MHC-I: major histocompatibility complex class I
MPs-CLEIA: magnetic nanoparticle chemiluminescence enzyme immunoassay
MSI: microsatellite instability
mTORC1: mechanistic target of rapamycin complex 1
MVBs: multivesicular bodies
NK: natural killer
NSCLC: non-small cell lung cancer
PDAC: pancreatic ductal adenocarcinoma
P-gp: plasma membrane multidrug resistance efflux pump
PHD: prolyl hydroxylase
PI3K: phosphatidylinositol 3-kinase
PMN: pre-metastatic niche
PPI: protein-protein interaction
PPRP: proline-rich motif
PTM: post-translational modification
ROS: reactive oxygen species
SLC3A2: solute carrier family 3 member 2
TCA: tricarboxylic acid
TKI: tyrosine kinase inhibitor
TM3: third transmembrane helix
TMB: tumor mutational burden
TME: tumor microenvironment
TNBC: triple-negative breast cancer
ATPase: vacuolar H+-ATPase
VHL: von Hippel-Lindau
