# Supplementary material for: LAPTM4B in tumor immune evasion: lysosomal autophagy, metabolic adaptation, and therapeutic resistance
Source: Front Immunol. 2026 Sep 30;17:1946009. doi: 10.3389/fimmu.2026.1946009 (PMC13662648; doi:10.3389/fimmu.2026.1946009)
Supplement: Supplementary file 1 [file Table1.docx]

| Core Fields of Translational Medicine | Specific Technological Means and Assessment Strategies | Core Translational Mechanisms and Biochemical Network Remodeling | Clinical Guidance Significance and Intervention Prospects |
| --- | --- | --- | --- |
| Non-invasive Biopsy and Dynamic Monitoring | Dynamic monitoring of peripheral blood and serum biomarkers | Targeted sensing of the dynamic secretion features of LAPTM4B in bodily fluids | "Serum concentration is highly consistent with adjuvant therapy response, which can pre-warn breast cancer resistance signals earlier than imaging; LAPTM4B-35 combined with CA19-9 can greatly improve the early screening accuracy for occult pancreatic cancer (PDAC) [56, 58]" |
|  | Quantitative tracking system for exosomes | Tumors actively package highly oncogenic LAPTM4B-35 into exosomes and eject them into the systemic circulation | "Serving as long-range communication vectors, this can be used to quantitatively evaluate systemic micrometastasis risk and pre-warn the construction of the distant ""pre-metastatic niche (PMN)"" [54, 57]" |
|  | Highly sensitive detection of trace amounts in urine | Utilizing magnetic nanoparticle chemiluminescence enzyme immunoassay (MPs-CLEIA) to targetedly capture trace signals | "Creates highly sensitive, non-invasive precision screening conditions for urological tumors [59]" |
| In Vivo Capture and Non-invasive Imaging | Highly specific capture of circulating tumor cells (CTCs) | "Utilizing peptide biomimetic nano-interfaces targeting its extracellular loop, thoroughly breaking away from traditional EpCAM dependence" | "Capable of accurately capturing highly invasive cells undergoing epithelial-mesenchymal transition (EMT), supporting downstream single-cell sequencing and evolutionary mutation tracking [60]" |
|  | Non-invasive targeted molecular in vivo tracking | "Novel 18F-labeled PET probes ([18F]FP-LAP2H) overcome the background noise of glucose metabolism probes" | Achieves high signal-to-noise ratio and three-dimensional non-invasive visual tracking of specifically highly expressed lesions [61] |
|  | Targeted fluorescence navigation for surgical procedures | "Far-infrared/near-infrared fluorescent probes (e.g., DBT-2EEGIHGHHIISVG) emit intense fluorescence instantly upon encountering hydrophobic microenvironments" | "Lays the foundation for intraoperative real-time navigation for precise, sub-millimeter tumor lesion resection [64]" |
| Precision Dismantling of Multidimensional Resistance Barriers | Sensitization of traditional cytotoxic chemotherapy | Blocking the forced protonation and trapping of weakly basic drugs within highly acidic lysosomes; dismantling physical chimerization with the plasma membrane efflux pump (P-gp) | "Destroys the lysosomal drug trapping barrier in physical space, restoring the nuclear entry path and cytotoxicity of drugs such as doxorubicin and paclitaxel [3, 31]" |
|  | Synergistic targeted intervention with EGFR-TKIs | "Blocking the explosive accumulation of ATP1A1 and excessive vesicular acidification caused by TRIM8 degradation inhibition, restoring the degradation rate of endocytosed EGFR" | "Eliminates the continuous pro-proliferative signals sent from the endosomal membrane, significantly synergistically enhancing the sensitivity of non-small cell lung cancer to EGFR-TKIs [62]" |
|  | Parallel defense dismantling for radiotherapy and ferroptosis | Blocking the radiation-protective autophagic flux mediated by inactive EGFR-Beclin1; restoring the normal degradation of the antioxidant transporter SLC7A11 by the E3 ligase NEDD4L | "Closes the dual parallel defenses (anti-apoptotic and anti-ferroptotic networks) helping cancer cells survive lethal windows, thoroughly remodeling radiotherapy sensitivity [50, 63]" |
| Immune Stratification and Multidimensional Translational Paradigms | Pre-warning stratification of the local tumor immune microenvironment (TME) | "Combining bodily fluid LAPTM4B abundance, peripheral blood lactate levels, and the spatial infiltration features of tissue MDSCs to construct a composite clinical evaluation model" | "Precisely identifies high-risk populations with a risk of ""metabolic off-target"" responses to immune checkpoint inhibitors (ICIs), guiding them early into targeted joint metabolic intervention tracks" |
|  | Frontier targeted protein degradation (TPD) for transmembrane proteins | "Utilizing bifunctional LYTACs (e.g., the Apt-LYTAC conjugate system) to forcibly trigger clathrin-mediated endocytosis, dragging it into the lysosome for irreversible pulverization" | "Breaks the traditional ""undruggable"" barrier resulting from the lack of a kinase pocket, directly erasing this biochemical hub at the underlying physical level [69]" |
|  | Small-molecule allosteric antagonism and LNP-targeted delivery | "First-in-class compound ETS identifies and blocks Tyr285 phosphorylation and PI3K interaction of the oncogenic isoform; delivery of tumor-suppressive miRNAs (e.g., miR-188-5p, miR-489) blocks targeted translation" | "Instantly paralyzes PI3K/AKT pro-survival cascade signaling from the source and deeply curbs tumor metastasis progression [66, 67]" |
|  | """Metabolism-Lactate-Immunity"" joint blockade network" | "Applying MCT1/4 inhibitors (e.g., AZD3965) to deeply seal acid efflux channels, blocking the abnormal ""lactylation"" immune suppressive polarization of host immune cells from the epigenetic source" | "Reverses the microenvironmental acidosis chassis, generating potent synergy combined with anti-PD-1 therapies, promoting the transformation of suppressive cold tumors into inflammatory hot tumors, and breaking the curability bottleneck of multidrug-resistant solid tumors [13, 40]" |
