# Supplementary material for: LAPTM4B in tumor immune evasion: lysosomal autophagy, metabolic adaptation, and therapeutic resistance
Source: Front Immunol. 2026 Sep 30;17:1946009. doi: 10.3389/fimmu.2026.1946009 (PMC13662648; doi:10.3389/fimmu.2026.1946009)
Supplement: Supplementary file 2 [file Table2.docx]

| Tumor System Classification | Specific Cancer Type / Subtype | Upstream Genetic, Epigenetic Modification, and Non-coding RNA Regulatory Network | Core Downstream Protein Interactions, Signaling Cascades, and Cytological Phenotypes | Clinical Significance, Drug Resistance Mechanisms, and Microenvironmental Spatial Remodeling |
| --- | --- | --- | --- | --- |
| Digestive System | Hepatocellular Carcinoma (HCC) | Activated by HDAC2/AP4/ETV1 and SULF2 [1-4] | Activates YAP and WNT/β-catenin; increases CXCL8 secretion [5-6, 2] | Maintains stemness; drives MDSC-mediated PD-L1 resistance [2] |
|  | Colorectal Cancer (CRC) | Allele *2 amplification; driven by MYC [7-8] | Synergizes with LGR5 to maintain stemness [7] | LAPTM4B+/LGR5+ marks a high-risk stem-cell-like subtype with an extremely poor prognosis [7] |
|  | Gastric Cancer (STAD) | Upregulated by Helicobacter pylori infection [9] | Binds Beclin1, blocking the lysosomal degradation of EGFR [10] | Drives EMT; associated with lymph node metastasis under low RORα [9, 11] |
|  | Pancreatic Cancer (PDAC) | Chromosomal amplification; regulated by aberrant glycosylation modifications | Mediates the assembly and secretion of exosomes | Serum LAPTM4B-35 combined with CA19-9 improves early diagnosis rate (AUC=0.903) [12] |
| Respiratory System | Non-Small Cell Lung Cancer (NSCLC) | Upregulated via the TP73-AS1/miR-27b-3p axis [13] | Stabilizes ATP1A1 and SLC7A11 by blocking ubiquitination [14-15] | Induces EGFR-TKI resistance through lysosomal acidification; blocks ferroptosis [14-15] |
| Gynecological and Genitourinary Systems | Breast Cancer (BRCA) | Activated by AP4/CREB1 and upregulated by miR-132-3p loss [16-18] | Activates PI3K-AKT-mTOR through the PPRP motif | LAPTM4B copy-number gain is associated with inferior response to anthracycline-based chemotherapy in HR-negative tumors [19] |
|  | Ovarian Cancer and Cervical Cancer | Gene amplification and stress induction | Stabilizes HIF-1α; activates the VEGF/CDK12 network [20] | Promotes angiogenesis/invasion; an independent marker for chemoresistance [20-21] |
|  | Prostate Cancer (PRAD) | Upregulated by miR-188-5p loss [22] | Activates PI3K/AKT to resist chemotherapy stress [22] | Predicts resistance to doxorubicin and 6-AA [23] |
|  | Clear Cell Renal Cell Carcinoma (ccRCC) | Enriched in highly heterogeneous/metastatic subpopulations | Regulates chemokine-mediated intercellular communication | High LAPTM4B expression niches attract M2 macrophages and pro-metastatic stroma [24] |
| Skeletal, Skin, and Nervous Systems | Osteosarcoma (OS) | Accumulates due to miR-137 loss [25] | Stabilizes RhoA to activate the RhoA-LIMK-Cofilin axis [26] | Drives bone metastasis by promoting stress fiber assembly [25-26] |
|  | Malignant Melanoma | Upregulated via the SNHG16/miR-132 axis [27] | Maintains pro-survival and anti-apoptotic networks | Accelerates proliferation and dermal invasion [27] |
|  | Glioblastoma Multiforme (GBM) | Regulated by aberrant global mRNA methylation | Promotes the activation of the pAkt survival pathway | Increases microvascular density; indicates a sharp decline in OS [28] |
| Head and Neck, and Endocrine Systems | Papillary Thyroid Carcinoma (PTC) | Allele *2 repeats lead to stop codon readthrough [29] | Generates highly oncogenic N-terminal extended variants | A key risk factor for PTC onset and metastasis in females [29] |
|  | Head and Neck Squamous Cell Carcinoma / Nasopharyngeal Carcinoma | Induced by genotoxic stress such as radiation | Stabilizes inactive EGFR and Beclin1 [30] | Drives radiation-protective autophagy; associated with post-radiotherapy recurrence [30-32] |
| Hematological Malignancies | Acute Myeloid Leukemia (AML) | Evi1/Kdm6b erases H3K27me3 to unlock transcription [33] | Activates the LAPTM4B-mTOR axis; stabilizes RPS9 to activate STAT3 [33-34] | Promotes stem cell expansion; induces TKI cross-resistance [33-34] |
|  | Acute Lymphoblastic Leukemia (ALL) | Specifically enriched in leukemia-initiating cells | Maintains malignant lymphocyte cycles | Knockout causes G0/G1 phase arrest and prolongs survival [35] |

**References**

[1] M. Wang, J. Liao, J. Wang, M. Xu, Y. Cheng, L. Wei, A. Huang, HDAC2 promotes autophagy-associated HCC malignant progression by transcriptionally activating LAPTM4B, Cell Death Dis 15(8) (2024) 593.

[2] H. Wang, Q. Zhou, D.F. Xie, Q. Xu, T. Yang, W. Wang, LAPTM4B-mediated hepatocellular carcinoma stem cell proliferation and MDSC migration: implications for HCC progression and sensitivity to PD-L1 monoclonal antibody therapy, Cell Death Dis 15(2) (2024) 165.

[3] Y. Meng, L. Wang, J. Xu, Q. Zhang, AP4 positively regulates LAPTM4B to promote hepatocellular carcinoma growth and metastasis, while reducing chemotherapy sensitivity, Mol Oncol 12(3) (2018) 373-390.

[4] Y. Ha, Y. Fang, P.A. Romecin Duran, E.J. Tolosa, C.D. Moser, M.E. Fernandez-Zapico, L.R. Roberts, Induction of Lysosome-associated Protein Transmembrane 4 Beta via Sulfatase 2 Enhances Autophagic Flux in Liver Cancer Cells, Hepatol Commun 3(11) (2019) 1520-1543.

[5] J. Wang, J. Liao, Y. Cheng, M. Chen, A. Huang, LAPTM4B enhances the stemness of CD133(+) liver cancer stem-like cells via WNT/β-catenin signaling, JHEP Rep 7(4) (2025) 101306.

[6] J. Liao, J. Wang, Y. Xu, Y. Wu, M. Wang, Q. Zhao, X. Tan, Y. Meng, L. Wei, A. Huang, LAPTM4B-YAP loop feedback amplification enhances the stemness of hepatocellular carcinoma, iScience 26(6) (2023) 106754.

[7] Y. Fang, T. Fu, Z. Xiong, Q. Zhang, W. Liu, K. Yu, A. Le, Novel Lysosomal-Associated Transmembrane Protein 4B-Positive Stem-Like Cell Subpopulation Characterizes High-Risk Colorectal Cancer Subtypes, MedComm (2020) 6(7) (2025) e70284.

[8] X.J. Cheng, W. Xu, Q.Y. Zhang, R.L. Zhou, Relationship between LAPTM4B gene polymorphism and susceptibility of colorectal and esophageal cancers, Ann Oncol 19(3) (2008) 527-32.

[9] S. Zhou, H. Chen, P. Yuan, N. Shi, X. Wang, J. Hu, L. Liu, Helicobacter pylori infection promotes epithelial-to-mesenchymal transition of gastric cells by upregulating LAPTM4B, Biochem Biophys Res Commun 514(3) (2019) 893-900.

[10] M. Tian, Y. Chen, D. Tian, X. Qiao, Z. Ma, J. Li, Beclin1 antagonizes LAPTM4B-mediated EGFR overactivation in gastric cancer cells, Gene 626 (2017) 48-53.

[11] S.N. El Mashad, M. Kandil, T.A.E. Talab, A.E.N. Saied Abd El Naby, M.M. Sultan, A. Sohaib, A.S. Hemida, Gastric Carcinoma with low ROR alpha, low E- Cadherin and High LAPTM4B Immunohistochemical Profile; is associated with unfavorable prognosis in Egyptian patients, J Immunoassay Immunochem 45(1) (2024) 50-72.

[12] Z. Yang, N. Senninger, I. Flammang, Q. Ye, S.A. Dhayat, Clinical impact of circulating LAPTM4B-35 in pancreatic ductal adenocarcinoma, J Cancer Res Clin Oncol 145(5) (2019) 1165-1178.

[13] Q. Jiang, W. Xing, J. Cheng, Y. Yu, Long Non-Coding RNA TP73-AS1 Promotes the Development of Lung Cancer by Targeting the miR-27b-3p/LAPTM4B Axis, Onco Targets Ther 13 (2020) 7019-7031.

[14] R. Yan, D. Liu, H. Guo, M. Liu, D. Lv, B. Björkblom, M. Wu, H. Yu, H. Leng, B. Lu, Y. Li, M. Gao, T. Blom, K. Zhou, LAPTM4B counteracts ferroptosis via suppressing the ubiquitin-proteasome degradation of SLC7A11 in non-small cell lung cancer, Cell Death Dis 15(6) (2024) 436.

[15] D. Liu, M. Liu, D. Lv, Y. Li, H. Guo, B. Lu, H. Leng, R. Yan, H. Yu, T. Blom, K. Zhou, LAPTM4B Confers Resistance to EGFR-TKIs by Suppressing the Proteasomal Degradation of ATP1A1 in Non-small Cell Lung Cancer, Int J Biol Sci 22(1) (2026) 1-24.

[16] L. Wang, Y. Meng, J.J. Xu, Q.Y. Zhang, The Transcription Factor AP4 Promotes Oncogenic Phenotypes and Cisplatin Resistance by Regulating LAPTM4B Expression, Mol Cancer Res 16(5) (2018) 857-868.

[17] M. Zhang, J.J. Xu, R.L. Zhou, Q.Y. Zhang, cAMP responsive element binding protein-1 is a transcription factor of lysosomal-associated protein transmembrane-4 Beta in human breast cancer cells, PLoS One 8(2) (2013) e57520.

[18] S. Li, J.J. Xu, Q.Y. Zhang, MicroRNA-132-3p inhibits tumor malignant progression by regulating lysosomal-associated protein transmembrane 4 beta in breast cancer, Cancer Sci 110(10) (2019) 3098-3109.

[19] O. Rusz, O. Papp, L. Vizkeleti, B.A. Molnar, K.C. Bende, G. Lotz, B. Acs, Z. Kahan, T. Szekely, A. Bathori, C. Szundi, J. Kulka, Z. Szallasi, A.-M. Tokes, LAPTM4B gene copy number gain is associated with inferior response to anthracycline-based chemotherapy in hormone receptor negative breast carcinomas, Cancer Chemother Pharmacol 82(1) (2018) 139-147. doi: 10.1007/s00280-018-3602-z.

[20] F. Meng, X. Chen, H. Song, G. Lou, LAPTM4B down regulation inhibits the proliferation, invasion and angiogenesis of HeLa cells in vitro, Cell Physiol Biochem 37(3) (2015) 890-900.

[21] M. Yin, C. Li, X. Li, G. Lou, B. Miao, X. Liu, F. Meng, H. Zhang, X. Chen, M. Sun, Q. Ling, R. Zhou, Over-expression of LAPTM4B is associated with poor prognosis and chemotherapy resistance in stages III and IV epithelial ovarian cancer, J Surg Oncol 104(1) (2011) 29-36.

[22] H. Zhang, S. Qi, T. Zhang, A. Wang, R. Liu, J. Guo, Y. Wang, Y. Xu, miR-188-5p inhibits tumour growth and metastasis in prostate cancer by repressing LAPTM4B expression, Oncotarget 6(8) (2015) 6092-104.

[23] J. Cheng, D. Mao, Prediction of Prostate Adenocarcinoma Recurrence Prognosis and Immune Status Through 6-Acetoxy-Anopterine Resistance-Associated Programmed Cell Death Genes, Cancer Manag Res 18 (2026) 552328.

[24] H. Wang, Q. Wang, Y. Wu, J. Lou, S. Zhu, Y. Xu, Autophagy-related gene LAPTM4B promotes the progression of renal clear cell carcinoma and is associated with immunity, Front Pharmacol 14 (2023) 1118217.

[25] R. Yan, D. Liu, J. Wang, M. Liu, H. Guo, J. Bai, S. Yang, J. Chang, Z. Yao, Z. Yang, T. Blom, K. Zhou, miR-137-LAPTM4B regulates cytoskeleton organization and cancer metastasis via the RhoA-LIMK-Cofilin pathway in osteosarcoma, Oncogenesis 12(1) (2023) 25.

[26] Z.X. Wang, M.Y. Guo, J. Ren, G.S. Li, X.G. Sun, Identification of Lysosome-Associated Protein Transmembrane-4 as a Novel Therapeutic Target for Osteosarcoma Treatment, Orthop Surg 12(4) (2020) 1253-1260.

[27] L.L. Bi, X.Q. Hua, W.H. Li, L. Wang, Y. Li, X.F. Jia, SNHG16 promotes cell proliferation and migration through sponging miR-132 in melanoma, J Biol Regul Homeost Agents 34(4) (2020) 1307-1316.

[28] X. Dong, K. Tamura, D. Kobayashi, N. Ando, K. Sumita, T. Maehara, LAPTM4B-35 is a novel prognostic factor for glioblastoma, J Neurooncol 132(2) (2017) 295-303.

[29] Y. Meng, R. Zhou, J. Xu, Q. Zhang, LAPTM4B*2 allele is associated with the development of papillary thyroid carcinoma in Chinese women, Oncol Lett 14(3) (2017) 3421-3428.

[30] C. Chu, X. Niu, X. Ou, C. Hu, LAPTM4B knockdown increases the radiosensitivity of EGFR-overexpressing radioresistant nasopharyngeal cancer cells by inhibiting autophagy, Onco Targets Ther 12 (2019) 5661-5677.

[31] U. Kotowski, L. Kadletz, S. Schneider, F. Oberndorfer, J. Schnoell, E. Gurnhofer, L. Kenner, T. Lucas, G. Heiduschka, Overexpression of LAPTM4B-35 is a negative prognostic factor in head and neck squamous cell carcinoma, Sci Rep 9(1) (2019) 18866.

[32] J. Fan, J. Yang, W. Qiao, W. Liu, C. Xing, LAPTM4B-35 expression is associated with pathological grades and clinical stages in salivary adenoid cystic carcinoma, Oncol Lett 19(1) (2020) 317-322.

[33] Q. Wu, C. Yu, F. Yu, Y. Guo, Y. Sheng, L. Li, Y. Li, Y. Zhang, C. Hu, J. Wang, T.C. He, Y. Huang, H. Ni, Z. Huo, W. Wu, G.G. Wang, J. Lyu, Z. Qian, Evi1 governs Kdm6b-mediated histone demethylation to regulate the Laptm4b-driven mTOR pathway in hematopoietic progenitor cells, J Clin Invest 134(24) (2024). doi: 10.1172/JCI173403.

[34] Y. Huang, M. Peng, H. Qin, Y. Li, L. Pei, X. Liu, X. Zhao, LAPTM4B promotes AML progression through regulating RPS9/STAT3 axis, Cell Signal 106 (2023) 110623. doi: 10.1016/j.cellsig.2023.110623.

[35] H. Zhou, Y. Yi, W. He, L. Zheng, Y. Hu, T. Niu, A comprehensive prognostic and immune analysis of LAPTM4B in pan-cancer and Philadelphia chromosome-positive acute lymphoblastic leukemia, Front Immunol 16 (2025) 1522293.
